# Carcinosarcoma of the corpus uteri (Malignant Müllerian Mixed Tumor): a case report in Yaoundé (Cameroon)

**DOI:** 10.11604/pamj.2013.16.145.3497

**Published:** 2013-12-19

**Authors:** Zacharie Sando, Florent Ymele Fouelifack, Jovanny Tsuala Fouogue, Jeanne Hortence Fouedjio, Chimene Etonga Anoudem, Charlette Nangue

**Affiliations:** 1 Yaounde Gyneco-Obstetric and Pediatric Hospital, Cameroon; 2 Faculty of Medicine and Biomedical Sciences of the University of Yaounde 1, Cameroon; 3 Yaoundé Central Hospital, Cameroon

**Keywords:** Carcinosarcoma, uterus, endometrium, Cancer, Cameroon

## Abstract

Carcinosarcoma of the uterus is a rare tumor representing 2-4% of uterine malignancies. Its prognosis is poor with a 5 years survival rate of 10-30%. We report a first documented case of carcinosarcoma occurring in a 62 years old woman who presented with postmenopausal vaginal bleeding for one year. The preoperative biopsy of endometrium revealed a leiomyosarcoma. Total body Computerized Tomography (CT) Scan revealed a mass limited to the uterus without other abnormalities. We carried out a total abdominal hysterectomy with bilateral salpingo-oophorectomy. Post operative histology of the specimen found a carcinosarcoma. The patient underwent a course of radiotherapy and a total body CT Scan done eight months later revealed no signs of recurrence or metastasis. Clinicopathological aspects, treatment options and prognosis of this aggressive neoplasm are reviewed. We recommend practitioners to be aware of this lesion for an early diagnosis and appropriate treatment.

## Introduction

Carcinosarcomas (CS) also called Malignant Mixed Müllerian Tumors (MMMTs) are very rare and extremely aggressive tumors of the uterine corpus containing both malignant epithelial and malignant mesodermal elements originating from the same precursor cell [1]. They account for 2 - 4% of all uterine malignancies and for about half of uterine sarcomas [2]. A recent study in a reference hospital in Cameroon found an annual incidence of 2.6 [3]. This article presents our experience about a case we managed at the Yaounde Gyneco-Obstetric and Pediatric Hospital, a tertiary center in Cameroon.

## Patient and observation

Mrs MNO 61 years old, G10P9018 is a retired teacher, menopaused since 9 years. She was referred to out unit for better management of a leiomyosarcoma diagnosed in a secondary hospital one month earlier on a specimen of endometrial biopsy (curettage) indicated for painless mild per vaginal bleeding evolving for one year. She had her first menses at 14 years old; she is menopaused since 9 years and she has had no sex for the past 15 years; there is no notion of sexually transmitted infection she has never done a pap smear cervical cytology; there is no personal history of gynecological malignancy. There is no contact cervical bleeding.

She is G10P9018 with 9 normal deliveries and one first trimester spontaneous abortion. The last child is 24 years old. She used to breastfeed for 18 months. Our patient has essential high blood pressure diagnosed nine years ago and treated with indapamide without visceral complications. She underwent two eye surgeries for cataract. Her blood group is A rhesus positive, she has never received blood products. She has no known allergy and consumes neither alcohol nor tobacco. She has three siblings who are apparently well as their mother. Her father died after a road traffic accident. She has no family history of cancer.

On systemic enquiry we found a foul smelling vaginal discharge, no pelvic pain, no cough, no chest pain, no weight loss, no anorexia and no vomiting. Her general condition was good and the blood Pressure was 140/88 millimeters of mercury. The weight was 116 kilogrammes and the height 1.64 meter (body mass index of 43.13 kilogrammes / meter square). The respiratory rate was 18 cycles per minute, the temperature 37.3° Celsius. Cardiac, pulmonary and breast examination were normal. Abdomen circumference was 125 centimeters. Inspection under vaginal speculum revealed a necrotic and hemorrhagic mass of seven centimeters of diameter protruding from the cervix. Digital vaginal examination was not done to avoid contact bleeding. Digital exploration of the rectum revealed no abnormalities. Varicosities were found on both legs.

**Figure 1:**
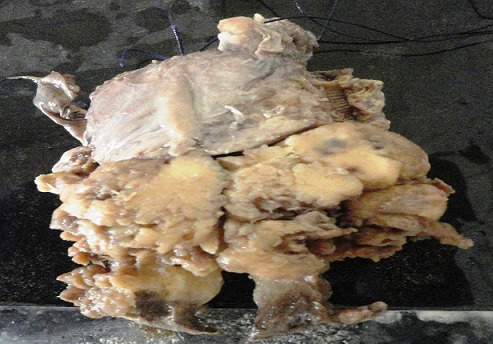
Macroscopy showing bulky and necrotic poorly circumcised mass of the corpus uterus

**Figure 2:**
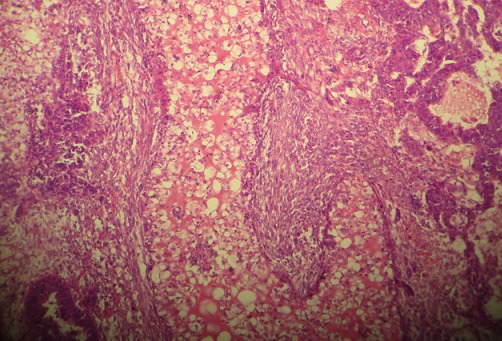
Micrograph showing an admixture of malignant epithelial elements (undifferentiated and squamous carcinoma) and mesenchymal elements (leiomyosarcoma)

The following work up was done: chest radiography revealed a heart of increased size but no pulmonary abnormality; Electrocardiogram revealed ventricular extra-systoles; ultrasonography and CT Scan of the abdomen and pelvic revealed a mass limited to the uterine cervix without others abnormalities. The full blood count, liver and function tests and hemostasis work up were normal. Hepatitis B and Human Immunodeficiency Viruses test were negative. Fasting blood sugar level was normal. The working diagnosis was a uterine leiomyosarcoma at stage II of the FIGO (International Federation of Obstetricians and Gynecologists) staging system. A total abdominal hysterectomy with bilateral salpingo-oophorectomy was carried out under general anesthesia. The findings were: normal size uterus containing a seven centimeters mass prolapsed through the cervix; uterine serosa invaded by the tumor; macroscopically normal adnexae. The pelvis was free of adhesions. The post operative course was complicated by infection of the abdominal wall. The abscess was drained and secondary closure of the wound was done 22 days after surgery and the patient sent for adjuvant radiotherapy. The Macroscopy showed bulky and necrotic poorly circumcised mass of the corpus uterus (Figure 1). The Histopathology analysis with hematoxylin and eosin stain showed a neoplasm composed of admixture of malignant epithelial and mesenchymal elements. The epithelial element was non glandular mostly undifferentiated and squamous carcinoma. The mesenchymal elements were homologous, consisting of leiomyosarcoma. Features were consistent with a Mixed Malignant Müllerian Tumor (MMMTs) also called Carcinosarcoma of the corpus uteri with pathological stage PT3 (Figure 2).

## Discussion

Since carcinosarcomas are very rare their risk factors have not been widely studied. Nevertheless the following factors are known to increase the risk of having a carcinosarcoma: prior pelvic irradiation, chronic estrogen exposure, tamoxifen use and African-American race [1]. Our patient is african and was chronically exposed to estrogens because of her morbid obesity. She had neither been irradiated nor taken tamoxifen. Tobacco smoking and combined contraceptive pill are known as protective agents but were not found in our patient. CS can occur at any age but mean age at diagnosis varies between 65 and 71 year old [1, 4]. Our patient was 61 year old. The classical presentation of CS includes abnormal uterine bleeding, sensation of pelvic mass, pelvic discomfort or pain, constipation and urinary urgency [2, 4–5]. Our patient presented with twelve months post-menopausal bleeding. The relatively slow growth of her tumor may explain the absence of rectal and bladder symptoms; indeed the biggest dimension of the uterus was nine centimeters. There is no blood marker for CS and the gold standard diagnosis is histological. Specimen can be obtained by endometrial biopsy and/or hysterectomy, the later being more accurate and appropriate for staging. Imaging techniques are used to appreciate local extension of the tumor to guide surgical procedure or to preclude it in case of unresectable tumors. The second purpose of imaging techniques is to look for metastases. CT scan usually suffices, but magnetic resonance imaging and positron-emission tomography scanning are advocated by some authors for post-operative follow-up and for differentiating between CS and benign tumors [1, 2]. In our setting only CT scan is available and was obtained after many efforts by our patient to afford it. The first histological analysis identified a leimyosarcoma but it was done on a specimen of endometrial biopsy which sensitivity to detect CS is low [1, 2]. The final histological analysis, carried out on the specimen of total hysterectomy and bilateral salpingo-oophorectomy, concluded to CS. Though immuno-histo-chemical and molecular aspects may be helpful to establish the prognosis of CS [6, 7], they are not routinely determined in our resource-poor setting. The best management of CS is total hysterectomy which is usually enough. Bilateral salpingo-oophorectomy is recommended after menopause [2, 8] as we did in our case. Adjuvant pelvic radiotherapy and/or chemotherapy yield the same results in term of recurrence-free interval and five years survival rate [4, 9]. Prognosis factors of CS are: stage of tumor at diagnosis, tumor free resection margins, epithelial nuclear grade and depth of myometrial invasion. The overall five years survival rate of CS is 10 - 50% and when tumor is outside the uterus at the time of diagnosis the two years survival rate is less than 10% [2, 10]. In our case the tumor was at stage pT3b and the resection margins were invaded by malignant cells; moreover the nuclear grade was quoted at 3 giving a poor prognosis to our patient. Nevertheless, eight months after surgery and adjuvant radiotherapy she was alive and without evidence of tumor recurrence or disease progression.

## Conclusion

This CS has been managed according to international standards in our resource-poor setting with success. We recommend to practitioners and researchers to report other cases to increase awareness on mixed malignant mesodermal tumor.
